# Nonspecific symptoms explain functional limitations in older adults better than disease-specific symptoms

**DOI:** 10.1007/s41999-026-01583-y

**Published:** 2026-08-18

**Authors:** Sandra L. Wezeman, René J. F. Melis, Cillian Hourican, Rick Quax, Thomas M. Gill, Marcel G. M. Olde Rikkert, Geeske Peeters

**Affiliations:** 1 Department of Geriatric Medicine, Radboud University Medical Center, Nijmegen, The Netherlands; 2 Computational Science Lab, Institute of Informatics, University of Amsterdam, Amsterdam, The Netherlands; 3 Institute for Advanced Study, Amsterdam, The Netherlands; 4 Department of Internal Medicine, Yale University School of Medicine, New Haven, CT USA; 5 Radboudumc Alzheimer Centre, Radboud University Medical Centre, Nijmegen, The Netherlands

**Keywords:** Symptom burden, Activities of daily living, Functional independence, Longitudinal studies

## Abstract

**Purpose:**

Older adults often have multiple diseases and age-related physiologic changes that may present with nonspecific symptoms such as fatigue or dizziness. Identifying symptoms that are most strongly associated with functioning may guide symptom management. This study examined the association of nonspecific and disease-specific symptoms with functional limitations in community-dwelling older adults over a 23-year period.

**Methods:**

Data were from the Precipitating Events Project, a longitudinal cohort study, involving 754 adults aged 70 and older [mean (standard deviation) age of 78.9 (5.3) years, 64.6% female] from South Central Connecticut, USA. During monthly telephone interviews, 20 symptoms and limitations in 13 daily activities were assessed. Associations were cross-sectionally and longitudinally tested using trends in proportions (*Z*-scores) and generalized estimating equations models.

**Results:**

The three strongest trends in proportions with functional limitations were for the nonspecific symptoms: ‘memory problems’ (*Z*-score 60.9), ‘arm or leg weakness’ (58.5), and ‘fatigue’ (54.7). The lowest *z*-scores were for the disease-specific symptoms ‘cold or flu symptoms’ (12.3), ‘decreased eyesight’ (17.8), and ‘chest pain’ (19.9). The three nonspecific symptoms: ‘fall’ [regression coefficient *b* = 1.40, confidence interval (CI) 1.28–1.52], ‘arm or leg weakness’ (*b* = 1.13, CI 1.02–1.23), and ‘dizziness’ (*b* = 1.12, CI 1.04–1.20) had the strongest cross-sectional associations with functional limitations. In longitudinal models with a 1-month time lag, these associations attenuated.

**Conclusion:**

Nonspecific symptoms had stronger associations with functional limitations than disease-specific symptoms, underscoring the importance of addressing nonspecific symptoms in older adults. The presence of cross-sectional, but not longitudinal, associations likely reflects an immediate rather than sustained impact on functioning.

**Supplementary Information:**

The online version contains supplementary material available at https://doi.org/10.1007/s41999-026-01583-y.

## Key Points

**Aim:**

This study examined the association of disease-specific symptoms (e.g., chest or joint pain) and nonspecific symptoms (e.g., fatigue, dizziness, anxiety) with functional limitations in community-dwelling older adults over a 23-year follow-up.

**Findings:**

The nonspecific symptoms ‘fall or injury’, ‘arm or leg weakness’, and ‘dizziness’ demonstrated the strongest cross-sectional associations with functional limitations in community-dwelling older adults. Memory or thinking problems was the only symptom with a significant positive association with more functional limitations 1 month later, suggesting that most symptoms relate more closely to current than future daily functioning.

**Message:**

This study demonstrates that nonspecific symptoms often have stronger cross-sectional associations with functional limitations than most disease-specific symptoms, highlighting the need to assess nonspecific symptoms for a comprehensive understanding of the functional burden in older adults.

**Supplementary Information:**

The online version contains supplementary material available at https://doi.org/10.1007/s41999-026-01583-y.

## Introduction

The health of older adults is often affected by multiple symptoms and diseases. Older adults commonly have patterns of illness that manifest through nonspecific disease presentations rather than disease-specific signs and symptoms and therefore do not fit neatly into specific disease categories [1]. Consequently, disease-focused approaches often fall short when evaluating this complex interplay between symptoms and diseases [2]. For older adults with multiple chronic diseases, symptom or syndrome-focused guidance or interventions may add more value than treatment of individual diseases, a shift that is also relevant since disease-modifying therapies for many age-related chronic diseases are lacking [3]. This shift in focus aligns with the calls in geriatric medicine to move beyond the individual disease as the organizing focus of care for older adults [1, 4, 5]. Therefore, it can be more useful to focus on symptoms related to overall health and daily functioning than applying the traditional clinical approach of focusing merely on disease diagnoses [1, 6].

Understanding which symptoms have the greatest impact on health outcomes can be achieved by examining the relationship between symptoms and functional limitations. Identifying symptoms that are most strongly associated with functioning may guide clinicians to decide which symptom(s) to treat to achieve the greatest improvements in functioning [7]. If the presence of certain symptoms appears to be strongly associated with functional limitations, intervention strategies may be directed at these symptoms to reduce their negative consequences [8]. However, few studies have examined the impact of symptoms on functioning in the general population of community-dwelling older adults. Therefore, this study aims to investigate the associations of common physical and mental health symptoms with functional limitations in community-dwelling older adults [9–12].

In this paper, we differentiate between disease-specific and nonspecific symptoms. Symptoms were classified as specific when they can be referred to a single organ system or mechanism (e.g., joint pain to the musculoskeletal system, decreased eyesight to the visual system), regardless of how many diseases may cause them, and as nonspecific when their causes act through several interacting systems [13]. In contrast, nonspecific symptoms, such as fatigue and dizziness, often stem from the complex interaction of multiple organ systems and underlying pathologies [14]. These symptoms are often also described as (components of) a geriatric syndrome, characterized as a multifactorial symptom (cluster) that cannot be traced back to a singular disease, but instead reflects the simultaneous, interacting presence of several factors [4, 5, 13], including comorbidities, psychosocial factors, and age-related physiological changes [15, 16]. Geriatric syndromes arise when accumulated impairments across multiple organ systems leave an older person vulnerable to situational challenges [17]. Commonly occurring syndromes include falls, delirium, and urinary incontinence, which share interacting risk factors (e.g. older age, impaired mobility, cognitive impairment and functional impairment) rather than a single causal disease [4, 13]. These conditions are consistently associated with adverse outcomes, such as disability, lower quality of life, and mortality [4, 13, 17].

To understand why illnesses in older adults frequently manifest as geriatric syndromes or nonspecific symptoms, it is essential to consider the underlying concepts of intrinsic capacity and frailty [16]. Intrinsic capacity denotes the composite of an individual’s physical and mental capacities, the decline of which is thought to underlie the nonspecific manifestation of illness in later life [16, 18]. Frailty denotes diminished ability to withstand acute stressors [19]. Frail older adults are twice as likely to exhibit nonspecific disease presentations than non-frail older adults [20]. Indeed, in a study of older patients with COVID-19, those with a nonspecific presentation were more frail than those with a disease-specific presentation [21]. Since this same loss of reserves can be a driver of functional decline, we hypothesize that nonspecific symptoms act as clinical markers of impaired daily functioning. This underlying mechanism forms the premise of our study, leading us to investigate whether nonspecific symptoms are more strongly associated with functional limitations than disease-specific symptoms.

The presence of nonspecific symptoms or the absence of specific symptoms complicates the care for older adults [15, 20]. Nonspecific symptoms are associated with poor health outcomes including frequent hospitalizations, longer hospital stays, increased mortality, higher health care costs and most importantly unnecessary limitations in functioning [5, 14, 16, 20, 22]. In previous work, the presence of nonspecific symptoms, e.g., delirium, was predictive of failure to regain premorbid functional status in hospitalized patients [20]. Hospitalized patients with nonspecific symptoms were more often discharged to a long-term care institution, reflecting a decline in their health [5, 20]. Among community-dwelling older adults with advanced chronic disease, 86% experience at least one symptom [23], with fatigue and limited activity being reported most frequently [23, 24]. In a study of older adults with chronic diseases, fatigue disrupted usual recreational activities and everyday chores, directly affecting quality of life [25]. Fatigue limited exercise capacity, mobility, motivation and concentration [25]. Both specific and nonspecific symptoms can be disabling, for example chest pain in acute coronary syndromes, or a fall in a frail older adult. Yet whereas disease-specific symptoms are routinely tied to a diagnosis and treatment, nonspecific symptoms are frequently under-recognized despite their functional impact, which is why their association with functional limitations warrants study.

Therefore, this study investigates the associations of nonspecific and specific symptoms with functional limitations in community-dwelling older adults. We also examined whether symptoms were longitudinally associated with functional limitations reported 1 month later.

## Methods

We used data from the Precipitating Events Project (PEP) [26]. The PEP is an ongoing longitudinal cohort study of 754 community-dwelling older adults aged 70 years and older from South Central Connecticut, USA. This study was established to evaluate the epidemiology of symptoms and disability in older adults and elucidate the role of intervening illnesses in the disabling process [26]. The PEP study was approved by the Yale Human Investigation Committee, and all participants provided verbal informed consent [27]. The ethics committee of the Radboudumc considered the current secondary analysis of the PEP data to fall outside the remits of the Medical Research Involving Human Subjects Act (WMO), and therefore not subject to further ethical review.

### Participants

Potential participants were identified from a list of 3157 members aged 70 years and older of a large health plan in South Central Connecticut [26]. Eligibility was determined by a telephone interview and confirmed during a home visit [26]. Only 4.6% of individuals refused screening, and 75.2% of eligible persons agreed to participate [26]. Individuals who declined participation did not differ significantly from those who were enrolled in terms of age or sex [26]. Persons were potentially eligible if they were independent in four basic activities of daily living (ADL): bathing, dressing, walking around the house, and transferring from a chair [26]. Persons were excluded if they had significant cognitive impairment with no available proxy, diagnosis of a terminal illness with a life expectancy of less than 12 months, plans to move out of the area in the next 12 months, or inability to speak English [26].

Participants were enrolled in a 4:2:1 ratio for low, intermediate, and high risk for disability [12, 28, 29]. Someone was classified as low risk if he or she took 10 s or less to walk back and forth over a 10-foot (3 m) course as quickly as possible (rapid gait test). Participants who needed more than 10 s to complete the rapid gait test, were classified as intermediate risk if they were younger than 85 and scored 24 or higher on the Mini-Mental State Examination (MMSE), or high risk if they either scored less than 24 on the MMSE or were 85 years of age or older. More information on the stratified sampling is provided elsewhere [12, 28]. Participants were enrolled between March 1998 and October 1999. Up to April 2022, 727 (96.4%) participants had died after a median follow-up of 109 months, while 43 (5.7%) withdrew from the study after a median of 27 months.

### Data collection

Data used in our study were collected from 1998 to 2022. Participants completed monthly telephone interviews and comprehensive home assessments every 18 months (Table 1). During the home assessments, participants were asked about their psychosocial health behaviors e.g., self-reported health, social activities, smoking behavior, and data were collected about their body mass index (BMI), gait speed and cognition. Participants were interviewed monthly to ascertain symptoms and functional limitations.

**Table 1:** Overview of assessments, their timing, and instruments in the PEP study

Assessment (instrument)	Timing/frequency	Content assessed
Baseline home visit (standardized nurse assessment	Once, at enrollment (1998–1999)	Age, sex, ethnicity, education, marital and living status, smoking, height and weight (BMI), cognition (MMSE), frailty (Fried Frailty phenotype), number of chronic conditions, prescribed medications
Monthly telephone interview	Every month (1998–2022)	Restricted activity (2 items); presence of 20 symptoms and whether each restricted activity; help needed for 13 daily activities (range 0–13)
Comprehensive home assessment	Every 18 months	Self-reported health, social activities, smoking, BMI, gait speed, cognition

### Symptom assessment

The presence of the 20 symptoms listed in Table 2 was assessed by monthly telephone interviews. The list was assembled by the PEP investigators by identifying common physical and mental health symptoms that community-living older persons had reported in previous studies [9, 10], supplemented with additional symptoms judged important on the basis of clinical and research experience, such as falling and fear for falling [11, 12]. Participants were asked whether they had restricted their activity since the last interview. Restricted activity was defined as a ‘Yes’ to one or both of the following questions: (1) “Since we last talked, have you reduced your usual activities due to illness, injury, or other problem?”, and (2) “Since we last talked, have you stayed in bed for at least half a day due to illness, injury, or other problem?” [26]. If restricted activity was present, participants were asked about the presence of the 20 symptoms. For each reported symptom, participants were asked, “Did this symptom cause you to stay in bed for at least half a day or cut down on your usual activities?” [26]. If the answer to this question was yes, the symptom was labeled as a ‘restricting symptom’ and if the answer was ‘no’, the symptom was labeled as ‘non-restricting symptom’. Two datasets were made, one with only restricting symptoms and one dataset with restricting and non-restricting symptoms. We divided the symptoms into ‘specific’ and ‘nonspecific’ symptoms based on literature as listed in Table 2 [4, 5, 13, 30]. Ten symptoms are classified as specific, meaning that these mostly can be assigned to one organ system or disease mechanism, and predominantly have a monocausal etiology such as calf pain caused by peripheral leg ischemia. The other ten symptoms are classified as nonspecific, as they predominantly have a multifactorial origin [13].

**Table 2:** Subdivision nonspecific and specific symptoms in the PEP study

Nonspecific	Specific
Arm or leg weakness	Pain or stiffness in your joints
Been fatigued (no energy/very tired)	Pain or stiffness in your back
Been dizzy or unsteady on your feet	Leg pain on walking
A fall or injury	Swelling in feet or ankles
Been afraid of falling	Difficulty breathing or shortness of breath
Difficulty sleeping	Chest pain or tightness
Urinary incontinence	Poor or decreased eyesight
Been depressed	Frequent or painful urination
Been anxious or worried	Cold or flu symptoms
Memory or thinking problem	Nausea, vomiting, diarrhea, or stomach (abdominal) problem

### Functional limitations

Functional limitations were assessed by 13 questions about the daily activities of the participants. During the monthly interviews, participants were asked, “At the present time, do you need help from another person to (complete the task)?” for each of the four basic activities (bathing, dressing, walking across a room, and transferring from a chair), five instrumental activities (shopping, housework, meal preparation, taking medication, and managing finances), three mobility activities [walking a quarter mile (400 m), climbing a flight of stairs, lifting or carrying ten pounds (4.5 kg)] [26, 28, 31]. In addition, participants were asked “Have you driven a car during the past month?” [32]. For each activity they were unable to perform independently, one point was scored, adding up to a range of 0–13 functional limitations. These items are based on standard questions from well-established instruments for assessing basic and instrumental activities of daily living in older populations [28]. Multiple imputation was used to address the small amount (i.e., less than 1%) of missing data on functional limitations [33].

### Descriptive variables

During the first home visit, trained research nurses completed a comprehensive baseline assessment with standard instruments. Height and weight were measured and information about current and former smoking, marital and living situation, ethnicity and former education were assessed. The number of chronic conditions is the sum of 7 self-reported chronic diseases. These chronic diseases were heart attack, carcinoma, strokes, congestive heart disease, arthritis, chronic lung disease, and diabetes mellitus. Participants were classified as frail if they met 3 or more of the 5 criteria from the Fried phenotype [19]. These criteria included: self-reported weight loss more than 10 pounds in the past year, feeling exhausted most of the time during the previous week, low physical activity, low grip strength, and slow walking speed. Participants who met 1 or 2 of the criteria were classified as prefrail, and participants who met none of the criteria were considered non-frail [19]. The number of prescribed medications was assessed by review of pill bottles.

### Analyses

To investigate how the prevalence of symptoms varies per level of functional limitations (range 0–13), a Cochran–Armitage test for trend in proportions was used [34–36]. This cross-sectional analysis assessed population-level trends in symptom prevalence across all measurement time points. This test is suitable for analyzing trends in binomial proportions across ordered groups, but does not account for the correlation between repeated observations from the same individual. Data points were categorized into 14 groups based on functional limitation scores. For each of these groups, the proportion of data points on which a symptom was present was calculated. The test for trend in proportions provided the relative proportion of each symptom occurrence for each level of functional limitations. This test yields a *Z*-score to indicate whether there was a significant trend in symptom proportions across increasing levels of functional limitations.

A linear Generalized Estimating Equations (GEE) model was conducted to examine the longitudinal associations between time-varying symptoms and functional limitations. This method was chosen in addition to the test for trends in proportions to account for covariables and the within-person dependency of repeated measurements over time. To account for within-person dependency in the repeated monthly measurements, we selected an autoregressive working correlation structure over exchangeable and independent structures based on the best model fit (QIC). We evaluated the standard assumptions underlying linear GEE. As the symptom variables are dichotomous and the functional limitations outcome is continuous, their relationship inherently represents a mean difference, making linearity a given. The assumption of normally distributed residuals was confirmed through visual inspection of histograms and Q–Q plots. The GEE analyses used the full longitudinal dataset, incorporating all available symptom and functional limitation measurements obtained during follow-up and accounting for the correlation between repeated measurements within individuals. The associations of symptoms and functional limitations were investigated cross-sectionally and longitudinally. Specifically, the longitudinal associations were evaluated between symptoms measured in each month and functional limitations measured in the subsequent month. Both the cross-sectional and longitudinal analyses were adjusted for age, sex, ethnicity (White vs. Non-white), years of education and time-varying BMI. These covariables were selected based on existing literature and their associations with functional limitations [37, 38]. The longitudinal models were also adjusted for functional limitations measured at the same time point as the symptom. The analyses were not adjusted for chronic diseases, mood, or cognition as we believe these are mediators rather than solely confounders in the associations of interest.

Primary analyses were conducted using data from all participants with only symptoms that contributed to restricted activity as it was not known whether someone without restricted activity experienced symptoms. To explore the influence of non-restricting symptoms on the findings, sensitivity analyses were done including both restricting and non-restricting symptoms. All data analyses were performed using R (R 4.1.1).

## Results

754 participants had a mean (SD) age of 78.9 (5.3) years and 64.6% were female (Table 3). Most participants (90.5%) were White, and the average years of education was 12.0 (2.9). The median follow-up duration was 68 (interquartile range [IQR] 31–118) months. Averaged over time, the prevalence of the 20 restricting symptoms during the more than 23-year follow-up ranged from 1.4% for ‘frequent or painful urination’ to 11.1% for ‘fatigue’ (Table 4). Pooled across all individual observations during the entire follow-up period, the median number of functional limitations was 3 (IQR 1–7).

**Table 3:** Characteristics of participants enrolled in the PEP study at baseline

Characteristic	Total
*N*	754
Age, mean ± SD years	78.9 ± 5.3
Sex, *N* (%) woman	487 (64.6%)
Race/ethnicity, *N* (%) White	682 (90.5%)
Education, mean ± SD years	12.0 ± 2.9
Marital status, *N* (%) currently married	361 (47.9%)
Living status, *N* (%) living alone	298 (39.5%)
BMI, mean ± SD kg/m^2^	26.8 ± 5.2
Smoking, *N* (%)	
Current	63 (8.4%)
Former	417 (55.3%)
Never	274 (36.3%)
iADL dependent (0–4), median (IQR)	0 (0–1.0)
Frailty, *N* (%)	
Frail	194 (25.7%)
Pre-frail	386 (51.2%)
Not frail	174 (23.1%)
Number of prescribed medications, median (IQR)	4.0 (2.0–5.0)
Number of chronic conditions (0–7), median (IQR)	1.0 (0.0–2.0)

Participants were classified as frail if they met 3 or more of the 5 criteria from the Fried phenotype. These criteria included: self-reported weight loss more than 10 pounds in the past year, feeling exhausted most of the time during the previous week, low physical activity, low grip strength muscle weakness, and slow walking speed. Participants who met 1 or 2 of the criteria were classified as prefrail, and participants who met none of the criteria were considered as not frail [19]

*SD* standard deviation, *BMI* body mass index, *iADL* instrumental activities of daily living, *IQR* interquartile range

**Table 4:** Prevalence of restricting symptoms among participants enrolled in the PEP study during follow-up

Symptom	Prevalence (%)
*Fatigue*	*11.1*
Stiffness of joints	6.6
*Dizziness or unsteadiness*	*5.9*
Pain or stiffness in back	5.2
Shortness of breath	3.9
*Fear of falling*	*3.9*
Leg pain on walking	3.7
*Arm or leg weakness*	*3.6*
Nausea or vomiting or diarrhea	3.6
Cold or flu symptoms	3.4
Depression	3.1
*Sleeping problems*	*2.9*
*Anxiety or worry*	*2.7*
Swelling in feet or ankles	2.7
*Fall or injury*	*2.3*
*Memory or thinking problem*	*2.0*
*Urinary incontinence*	*1.8*
Chest pain or tightness	1.6
Poor or decreased eyesight	1.5
Frequent or painful urination	1.4

Rows with nonspecific symptoms are highlighted in italics

**Fig. 1:**
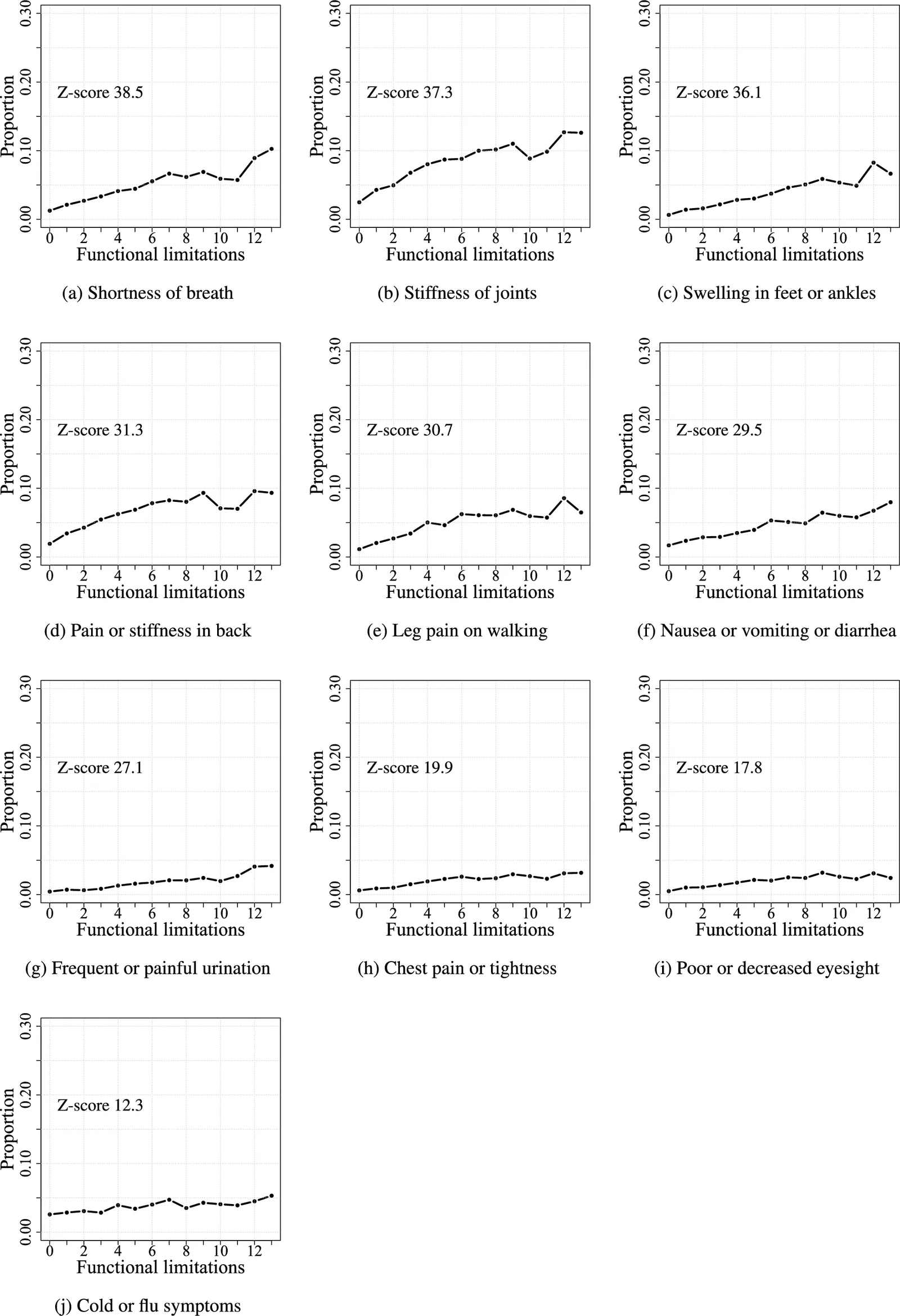
Trend in proportion of the specific symptoms and functional limitations. The figure displays the relative proportion (0–1) of each symptom occurrence (*y*-axis) across ascending levels (0–13) of functional limitations (*x*-axis). *Z*-scores indicate whether there was a significant trend in symptom proportions across increasing levels of functional limitations. All *Z*-scores were significant *ρ* ≤ 0.001

**Fig. 2:**
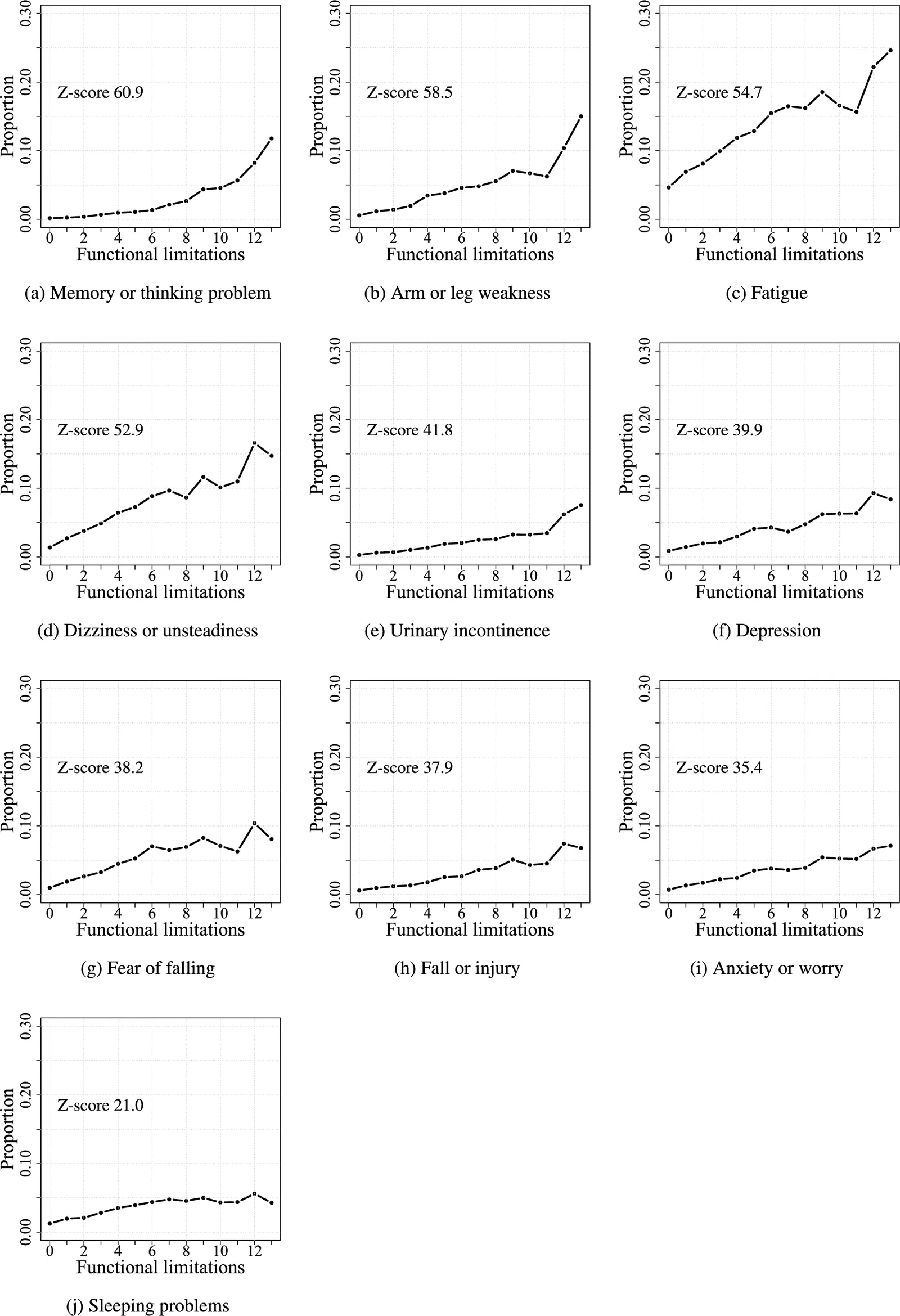
Trend in proportion of the nonspecific symptoms and functional limitations. The figure displays the relative proportion (0–1) of each symptom occurrence (*y*-axis) across ascending levels (0–13) of functional limitations (*x*-axis). *Z*-scores indicate whether there was a significant trend in symptom proportions across increasing levels of functional limitations. All *Z*-scores were significant *ρ* ≤ 0.001

All 20 restricting symptoms showed a significant cross-sectional trend with functional limitations. In other words, each symptom has a higher probability to be present at higher levels of functional limitations. The three highest *z*-scores—which imply the strongest trend in proportions of symptom presence across levels of functional limitations—were found in nonspecific symptoms, namely ‘memory or thinking problem’, ‘arm or leg weakness’ and ‘fatigue’ (Fig. 2a–c). The trends in proportions for specific and nonspecific symptoms with functional limitations are shown in, respectively, Figs. 1 and 2. The three lowest *z*-scores were found for the specific symptoms ‘cold or flu symptoms’, ‘decreased eyesight’ and ‘chest pain’ (Fig. 1h–j). The nonspecific symptoms ‘arm or leg weakness’, ‘fatigue’, and ‘dizziness’, were substantially more prevalent in older adults with a higher number of functional limitations compared to older adults with few functional limitations (Fig. 2b–d). The specific symptom ‘stiffness of joints’ also had a higher prevalence as the number of functional limitations increased (Fig. 1b). However, ‘chest pain’, ‘decreased eyesight’, ‘cold or flu symptoms’, and the nonspecific symptom ‘sleeping problems’ only had a slightly positive trend with functional limitations (Figs. 1h–j, 2j).

Supplementary Table S1 shows the *z*-scores of the trend in proportions of all reported restricting and non-restricting symptoms of participants who reported restricted activity. The addition of non-restricting symptoms resulted in lower *Z*-scores. ‘Memory or thinking problems’ and ‘arm or leg weakness’ still showed the strongest trends with functional limitations, ‘urinary incontinence’ now ranks the third highest.

In the cross-sectional GEE models, each symptom was significantly associated with functional limitations. Table 5 shows the results of the 20 GEE models with the regression coefficients in descending order for all symptoms. The three symptoms with the largest regression coefficients were the three nonspecific symptoms; ‘fall or injury’ (regression coefficient *b* = 1.40 points more functional limitations for present vs not present CI 1.28–1.52), ‘arm or leg weakness’ (*b* = 1.13, CI 1.02–1.23), and ‘dizziness’ (*b* = 1.12 CI 1.04–1.20).

**Table 5:** Associations between restricting symptoms and functional limitations among participants enrolled in the PEP study

Symptom	Cross-sectional coefficient (95% CI)	Longitudinal coefficient^a^ (95% CI)
*Fall or injury*	1.40 (1.28–1.52)***	− 0.17 (− 0.26 to − 0.08)***
*Arm or leg weakness*	1.13 (1.02–1.23)***	− 0.09 (− 0.15 to − 0.02)*
*Dizziness or unsteadiness*	1.12 (1.04–1.20)***	− 0.16 (− 0.21 to − 0.10)***
Swelling in the feet or ankles	0.99 (0.88–1.10)***	− 0.13 (− 0.21 to − 0.06)***
*Anxiety or worry*	0.90 (0.79–0.99)***	*− 0.05 (− 0.13–0.02)*
Frequent or painful urination	0.88 (0.73–1.03)***	− 0.05 (− 0.16–0.05)
Shortness of breath	0.88 (0.78–0.98)***	− 0.04 (− 0.11–0.02)
*Depression*	0.86 (0.77–0.96)***	*− 0.05 (− 0.11–0.02)*
*Sleeping problems*	0.86 (0.75–0.96)***	− 0.18 (− 0.26 to − 0.11)***
*Memory or thinking problem*	0.84 (0.72–0.97)***	0.21 (0.13–0.29)***
*Fatigue*	0.84 (0.78–0.90)***	− 0.12 (− 0.16 to − 0.09)***
Chest pain or tightness	0.82 (0.69–0.95)***	− 0.10 (− 0.20 to − 0.01)*
Nausea or vomiting or diarrhea	0.80 (0.70–0.90)***	− 0.14 (− 0.20 to − 0.07)***
*Fear of falling*	0.80 (0.71–0.88)***	− 0.11 (− 0.17 to − 0.05)***
Leg pain when walking	0.72 (0.64–0.81)***	− 0.09 (− 0.15 to − 0.03)**
Stiffness of joints	0.72 (0.65–0.80)***	− 0.11 (− 0.15 to − 0.06)***
Pain or stiffness in back	0.62 (0.54–0.69)***	− 0.04 (− 0.09–0.00)
*Urinary incontinence*	0.57 (0.44–0.69)***	*0.04 (− 0.05–0.12)*
Poor or decreased eyesight	0.45 (0.33–0.57)***	− 0.02 (− 0.12–0.08)
Cold or flu symptoms	0.40 (0.32–0.47)***	− 0.09 (− 0.15 to − 0.04)**

Results are ranked from the highest coefficient for the cross-sectional analyses to the lowest, and rows with nonspecific symptoms are highlighted in italics

Analyses were adjusted for age, sex, body mass index (BMI), ethnicity, and years of education

*CI* confidence interval

^***^ρ < 0.001, **ρ < 0.01, *ρ < 0.05

^a^Longitudinal coefficients show the association of symptoms with functional limitations 1 month later

In the longitudinal models, 13 symptoms were significantly associated with functional limitations in the subsequent month, of which 12 symptoms had negative, but low regression coefficients. ‘Memory or thinking problems’ was the only symptom with a statistically significant positive association (Table 5). Supplementary Table S2 shows the results of the sensitivity analyses including both restricting and non-restricting symptoms. Including non-restricting symptoms increased the prevalence of the symptoms. In these analyses, regression coefficients as well as the *Z*-scores of the trends in proportions were lower than in the primary analyses. However, across the 20 symptoms, the pattern persisted that nonspecific symptoms showed stronger cross-sectional associations with functional limitations than specific symptoms.

## Discussion

This study examined the association of nonspecific and specific symptoms with functional limitations. The prevalence of the nonspecific symptoms ‘memory or thinking problem’, ‘arm or leg weakness’, and ‘fatigue’ showed the steepest increase across rising levels of functional limitations. The three symptoms with the strongest associations with functional limitations were the nonspecific symptoms ‘fall or injury,’ ‘arm or leg weakness’, and ‘dizziness’. In the longitudinal models, these associations attenuated. Memory or thinking problems was the only symptom with a significant positive association with more functional limitations 1 month later.

We examined the prospective association over a 1-month interval to capture the short-term impact of symptoms on functioning, leveraging the study’s monthly data collection. Since symptoms are often transient and tend to resolve quickly, exploring longer lags might dilute the association as symptoms may have resolved within that timeframe. The stronger cross-sectional associations than longitudinal associations are understandable as symptoms relate more closely to current than future daily functioning and clearly have a low carry over effect to 1 month later. Because our longitudinal models adjust for functional limitations at the month of symptom assessment, coefficients can be interpreted as differences in next-month change. Negative longitudinal associations are to be explained by fluctuation of symptoms and improvement of functioning in the subsequent month. The reversal in the direction of associations shown in some symptoms likely reflects the transient nature of many symptoms. When a symptom resolves, the individual’s functional status often rebounds toward their baseline in the subsequent month. Therefore, while the immediate functional burden of a symptom is high (as captured cross-sectionally), it does not necessarily dictate a sustained functional decline once the symptom abates. However, certain symptoms (e.g., memory problems) have a less temporal dynamic than acute symptoms (e.g., cold or flu), which may explain their positive association with functional limitations 1 month later. The emergence of memory or thinking problems as the sole symptom with a positive association with functional limitations 1 month later is in line with evidence that cognitive symptoms exert a more sustained influence on functioning than transient somatic symptoms [39].

The two analytical approaches used in this study rank individual symptoms slightly differently because they evaluate distinct metrics. The test for trend in proportions assesses how steeply prevalence rises alongside functional limitations, whereas the GEE models estimate the adjusted mean difference. Nevertheless, both methods rank nonspecific symptoms the highest and specific symptoms the lowest, demonstrating that the main conclusion is robust to the chosen statistical method. The findings align with nonspecific disease presentation more broadly. For instance, a nonspecific presentation of COVID-19 was associated with greater frailty but did not independently predict worse outcomes (e.g., mortality) [21]. This is consistent with the premise that nonspecific symptoms act as markers of concurrent functional limitations rather than independent drivers of subsequent functional decline. Furthermore, Tinetti et al. reported that nonspecific symptoms contribute to health outcomes independently of specific diseases [7]. Situating the individual symptoms within the literature, the prominence of falls and fatigue is consistent with prior work linking these nonspecific symptoms to disability in older adults [11, 23–25, 40]. Relative to other conditions leading to hospitalization, falls were associated with worse disability outcomes and a higher likelihood of long-term nursing home admissions [40]. Similarly, symptom-burden studies in community-dwelling older adults identified fatigue as highly prevalent and bothersome [23–25].

A plausible explanation for the stronger cross-sectional associations of nonspecific symptoms with functional limitations than specific symptoms is that nonspecific symptoms affect daily functioning through different pathophysiological mechanisms. Whereas specific symptoms typically stem from localized pathology, nonspecific symptoms often arise from multiple pathophysiological mechanisms. As symptoms are the clinical manifestations of underlying pathology, the mechanisms through which multiple chronic diseases affect functioning likely apply to symptoms as well. Indeed, prior research indicates that combinations of diseases that affect daily functioning through different pathological mechanisms appear to be more limiting than combinations of diseases that arise from shared pathophysiologic mechanisms [41].

Nonspecific and specific symptoms had comparably low prevalences (Table 4), lower than those reported in community-dwelling adults in other studies [23, 24]. In older adults with advanced chronic diseases, prior research has shown that the prevalence was 81% for fatigue, 71% for shortness of breath, 32% for anxiety, and 22% for dizziness [24]. Individuals may tend to stop reporting highly prevalent symptoms or restricted activities when they consider them as chronic. However, a study of symptom burden found that almost all the participants who experienced symptoms of thinking problems, constipation, nausea, shortness of breath, pain, difficulty sleeping, or weakness (90–100%) considered them bothersome [24]. Between 65 and 84% of those reporting limited activity, fatigue, depressive symptoms, anxiety, and dizziness, also reported them as bothersome. These numbers may indicate that most of the experienced symptoms would be considered bothersome. However, when asked whether a symptom contributed to restricted activity, many symptoms were classified as non-restricting. This classification may help to indicate the severity of a symptom, in which restricting symptoms are more severe than symptoms that did not restrict the person in their activities. Nonetheless, we would advise to incorporate symptom severeness into future studies when investigating symptom burden.

Several limitations need to be considered when interpreting the findings. The reported prevalence of some symptoms was low. However, the large sample with a high frequency of repeated measurements ensured sufficient statistical power. Although several symptoms were rare in any given month, pooling repeated monthly observations over 23 years yielded estimates with narrow confidence intervals. It is important to bear in mind that the symptom occurrence was only assessed when someone experienced restricted activity in the past month. This question provides a standardized metric of severity, thereby enhancing clinical relevance of the symptom. However, this may have led to underreporting and underestimation of symptom prevalence. As such, the current prevalences are not representative of symptoms in general, but represent symptoms that lead to restricted activity. This may also explain the strong associations despite low prevalence. Another limitation is the lack of information on severity of both symptoms and functional limitations. Moreover, the unweighted sum of 13 daily activities does not capture the differential importance of individual activities (e.g., transferring from a chair versus driving or carrying weight). We could not account for interindividual differences or temporal fluctuation in symptom impact, since the same symptom may affect functioning differently depending on contextual factors, such as frailty or co-occurring symptoms. Limitations regarding the classification and operationalization of symptoms must also be acknowledged. The dichotomous categorization of symptoms into ‘specific’ and ‘nonspecific’, while grounded in geriatric syndrome literature, is inherently complex and requires further epidemiological validation regarding the multicausality criterion. For instance, although symptoms such as ‘urinary incontinence’ or ‘arm or leg weakness’ can occasionally be linked to a single organ system, they were classified as nonspecific in this study. This is because in the older population, they predominantly present as geriatric syndromes with multifactorial, interacting etiologies (e.g., medication side effects, mobility issues and cognitive impairment) rather than isolated pathology. Conversely, symptoms such as ‘leg pain on walking’ were classified as specific, as they typically reflect a distinct pathophysiological mechanism (e.g., vascular claudication). Furthermore, the eligibility criteria yielded a relatively more functionally independent and healthier cohort at enrollment. However, participants were followed for many years (median 109 months to death), during which a large proportion developed functional limitations. The long follow-up makes the sample more comparable to community-dwelling populations in daily functioning. In addition, the study population reported relatively few chronic conditions (Table 3) and thus represents comparatively healthy community-dwelling older adults. This limits comparability with, and generalizability to, more multimorbid populations, in whom symptom perception and impact may differ.

A major strength of this study was the possibility to investigate the impact of 20 symptoms on daily functioning over a long time in independently living older persons. Walke et al. suggest that instruments that assess 10 or fewer symptoms are likely to exclude some important symptoms [24]. Instruments that include 30 or more symptoms provide a comprehensive assessment but have a high respondent burden for older adults. Ideally, we would have had information on more symptoms (e.g., headaches, coughing, hearing problems), but to maintain a low response burden it is important to use a manageable list of symptoms to assess [24]. The data collection from the PEP study has several strengths, including the frequency of assessments, the long follow-up, the low attrition rate, and completeness of data [26]. The availability of data from monthly interviews for more than 23 years makes PEP a unique data source to study the association of symptoms with functioning. Another strength is the high participation and retention rate of participants. This makes the results generalizable to community-dwelling older adults independent in basic ADLs. Physically frail older adults were oversampled, resulting in a study population with a diverse distribution in range of frailty.

By focusing care on symptoms rather than primarily on diseases, symptom reduction and prevention can become a primary goal of care [2, 6]. Early recognition and management of symptoms may yield positive health outcomes, reduce the risk of new comorbidities, and improve quality of life [5]. Preventing and limiting the impact of disease on daily living in adults aged 70 and older requires interventions that include early identification and management of symptoms, especially those that mostly lead to functional limitations and ADL dependence [42]. Furthermore, prioritizing the symptoms most bothersome to older adults may facilitate a better balance between well-being and survival, ensuring that treatment targets remain aligned with patient priorities [4].

In conclusion, all symptoms were cross-sectionally associated with functional limitations, but not longitudinally. Nonspecific symptoms had stronger associations with functional limitations than most specific symptoms. The current findings highlight the importance of assessing nonspecific symptoms in understanding the functional burden in older adults aged 70 years and older.

## Supplementary Information

Below is the link to the electronic supplementary material.

Supplementary file1 (DOCX 29 KB)

## Data Availability

The data underlying this article were provided by the Department of Internal Medicine of the Yale University School of Medicine by permission.
