# Supplementary material for: Nonspecific symptoms explain functional limitations in older adults better than disease-specific symptoms
Source: Eur Geriatr Med. 2026 Aug 18;17(5):2509–20. doi: 10.1007/s41999-026-01583-y (PMC13755107; doi:10.1007/s41999-026-01583-y)
Supplement: Supplementary file 1 [file 41999_2026_1583_MOESM1_ESM.docx]

Supplementary information

# Aspecific Symptoms Explain Functional Limitations in Older Adults Better than Disease-specific Symptoms

Sandra L. Wezeman, MD, Msc^1^, René J.F. Melis, MD, PhD^1^, Cillian Hourican, Msc^2^, Rick Quax, PhD^2,3^, Thomas M. Gill, MD, PhD^4^, Marcel G.M. Olde Rikkert, MD, PhD^1,5^, Geeske Peeters, PhD^1,5^

1. Department of Geriatric Medicine, Radboud University Medical Center, Nijmegen, Netherlands
2. Computational Science Lab, Institute of Informatics, University of Amsterdam, Amsterdam, Netherlands
3. Institute for Advanced Study, Amsterdam, Netherlands
4. Department of Internal Medicine, Yale University School of Medicine, New Haven, CT, United States
5. Radboudumc Alzheimer Centre, Radboud University Medical Centre, Nijmegen, Netherlands

European Geriatric Medicine

Corresponding author: Geeske Peeters

E-mail address: Geeske.peeters@radboudumc.nl

# Supplementary Table S1. Trend in Proportions of Restricting and Non-restricting Symptoms by Number of Functional Limitations

| **Symptom** | **Z-score** | **p-value** |
| --- | --- | --- |
| Memory or thinking problem | 41.3 | <.001 |
| Arm or leg weakness | 39.5 | <.001 |
| Urinary incontinence | 34.9 | <.001 |
| Dizziness or unsteadiness | 22.2 | <.001 |
| Fall or injury | 20.7 | <.001 |
| Depression | 18.5 | <.001 |
| Fatigue | 17.6 | <.001 |
| Shortness of breath | 13.6 | <.001 |
| Swelling in the feet or ankles | 12.7 | <.001 |
| Fear of falling | 11.2 | <.001 |
| Anxiety or worry | 8.8 | <.001 |
| Nausea or vomiting or diarrhea | 7.9 | <.001 |
| Frequent or painful urination | 5.4 | <.001 |
| Leg pain when walking | 4.4 | <.001 |
| Poor or decreased eyesight | 3.9 | <.001 |
| Chest pain or tightness | 2.8 | .005 |
| Stiffness of joints | 1.5 | .130 |
| Pain or stiffness in the back | 1.0 | .298 |
| Sleeping problems | - 6.7 | <.001 |
| Cold or flu symptoms | - 9.5 | <.001 |

*Note:* Results are ranked from highest Z-score to lowest and rows with aspecific symptoms are highlighted in grey.

# Supplementary Table S2. Associations of Restricting and Non-restricting Symptoms with Functional Limitations

| **Symptom** | **Cross-sectional coefficient (95%CI)** | **p-value** | **Longitudinal coefficient ^a^ (95%CI)** | **p-value** |
| --- | --- | --- | --- | --- |
| Arm or leg weakness | 0.85 (0.73–0.96) | <.001 | 0.05 (-0.05–0.15) | .319 |
| Fall or injury | 0.81 (0.69–0.94) | <.001 | 0.06 (-0.05–0.17) | .318 |
| Dizziness or unsteadiness | 0.67 (0.57–0.77) | <.001 | -0.13 (-0.21– -0.06) | <.001 |
| Memory or thinking problem | 0.57 (0.45–0.70) | <.001 | 0.21 (0.12–0.30) | <.001 |
| Depression | 0.49 (0.38–0.60) | <.001 | 0.02 (-0.06–0.10) | .665 |
| Anxiety or worry | 0.45 (0.34–0.56) | <.001 | -0.09 (-0.17– -0.01) | .025 |
| Fatigue | 0.48 (0.37–0.59) | <.001 | -0.14 (-0.23– -0.05) | .002 |
| Shortness of breath | 0.43 (0.31–0.55) | <.001 | 0.02 (-0.06–0.10) | .660 |
| Chest pain or tightness | 0.36 (0.23–0.50) | <.001 | -0.18 (-0.30– -0.07) | .002 |
| Swelling in the feet or ankles | 0.34 (0.22–0.45) | <.001 | -0.03 (-0.10–0.05) | .452 |
| Urinary incontinence | 0.33 (0.21–0.45) | <.001 | 0.07 (-0.01–0.16) | .086 |
| Nausea, vomiting or diarrhea | 0.26 (0.16–0.36) | <.001 | -0.07 (-0.15–0.01) | .108 |
| Sleeping problems | 0.24 (0.13–0.35) | <.001 | -0.20 (-0.28– -0.11) | <.001 |
| Frequent or painful urination | 0.21 (0.11–0.32) | <.001 | -0.08 (-0.16–0.00) | .051 |
| Stiffness of joints | 0.15 (0.05–0.25) | .005 | -0.16 (-0.24– -0.09) | <.001 |
| Leg pain when walking | 0.13 (0.03–0.23) | .008 | -0.07 (-0.15–0.01) | .086 |
| Fear of falling | 0.09 (-0.01–0.20) | .083 | -0.05 (-0.13–0.03) | .230 |
| Pain or stiffness in back | 0.00 (-0.11–0.11) | .997 | -0.07 (-0.15–0.01) | .093 |
| Cold or flu symptoms | -0.16 (-0.25– -0.06) | .001 | -0.10 (-0.18– -0.01) | .028 |
| Poor or decreased eyesight | -0.17 (-0.28– -0.05) | .004 | 0.07 (-0.03– 0.16) | .161 |

*Note:* Results are ranked from highest coefficient for the cross-sectional analyses to lowest, and rows with aspecific symptoms are grey.

Analyses adjusted for age, sex, BMI, ethnicity (White vs. Non-white) and years of education.

CI, Confidence interval

^a^ Longitudinal coefficients show the association of symptoms with functional limitations one month later.
